# Development and Characterization of Biocompatible Chitosan-Aloe Vera Films Functionalized with Gluconolactone and Sorbitol for Advanced Wound Healing Applications

**DOI:** 10.1021/acsami.5c00715

**Published:** 2025-02-25

**Authors:** Beata Kaczmarek-Szczepańska, Patrycja Glajc, Dorota Chmielniak, Klaudia Gwizdalska, Maria Swiontek Brzezinska, Katarzyna Dembińska, Ambika H. Shinde, Magdalena Gierszewska, Krzysztof Łukowicz, Agnieszka Basta-Kaim, Ugo D’Amora, Lidia Zasada

**Affiliations:** † Department of Biomaterials and Cosmetics Chemistry, Faculty of Chemistry, Nicolaus Copernicus University in Torun, Gagarina 7, 87-100 Torun, Poland; ‡ Department of Environmental Microbiology and Biotechnology, Faculty of Biological and Veterinary Sciences, Nicolaus Copernicus University in Torun, Lwowska 1, 87-100 Torun, Poland; § Department of Physical Chemistry and Polymer Physical Chemistry, Faculty of Chemistry, Nicolaus Copernicus University in Torun, Gagarin 7, 87-100 Torun, Poland; ∥ Department of Experimental Neuroendocrinology, Laboratory of Immunoendocrinology, Maj Institute of Pharmacology, PolishAcademy of Sciences, 12 Smętna St., 31-343 Kraków, Poland; ⊥ Institute of Polymers, Composites and Biomaterials, National Research Council, v.le J.F. Kennedy 54, Mostra d’Oltremare, Pad. 20, 80125 Naples, Italy

**Keywords:** wound dressing, bioactivation, chitosan, aloe vera, gluconolactone, bioactive properties

## Abstract

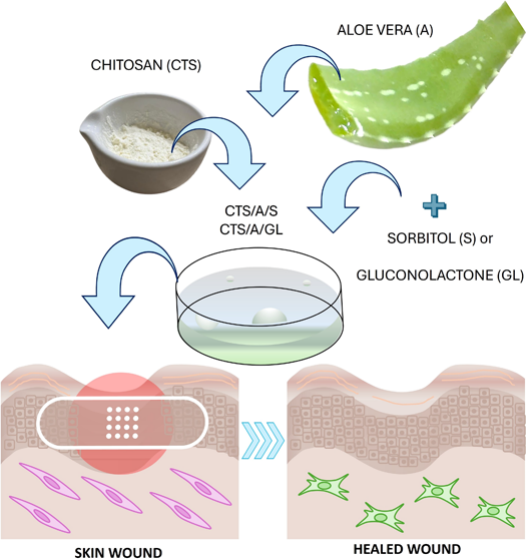

Chitosan (CTS) has emerged as a promising biopolymer for wound healing due to its biocompatibility, biodegradability, and intrinsic bioactive properties. This study explores the development and characterization of CTS-based films enhanced with natural bioactive agents, aloe vera (A), gluconolactone (GL), and sorbitol (S), to improve their mechanical, antimicrobial, and regenerative performance for potential use in advanced wound care. A series of CTS-based films were fabricated with varying concentrations of A, GL, and S, and their physicochemical, mechanical, and biological properties were comprehensively evaluated. Fourier transform infrared (FTIR) spectroscopy and atomic force microscopy (AFM) analysis revealed modifications in the film structure attributable to these additives, influencing the surface roughness, hydrophilicity, and thermal stability. Biocidal assays confirmed enhanced antimicrobial activity, particularly in films containing GL and A. Biodegradation studies demonstrated a significant enhancement in microbial decomposition of the films, while cytocompatibility tests confirmed minimal cytotoxic effects and improved cellular response. This research underscores the potential of combining CS with A, GL, and S to engineer multifunctional biomaterials tailored for effectively tackling different phases of the wound healing process, offering a sustainable and biocompatible alternative for clinical applications.

## Introduction

Over the past decades, many scientists have concentrated their research on developing advanced skin wound treatments to address a strong clinical and social need.^1−3^ These efforts aim at lowering medical expenses, offering lasting comfort, and encouraging efficient scar healing. Indeed, regenerative wound therapy, a rapidly evolving field in biomedical research, seeks to repair injured skin tissue and cells without leaving scars. By utilizing biomaterials, bioactive molecules, and cells, this approach strives to restore the skin to its original state, overcoming the limitations of conventional treatments that often result in scars, which can affect both aesthetics and physiological function.^4,5^ In this scenario, the advancement of biomaterials science and engineering has boosted the development of numerous skin substitutes, in many different forms, such as hydrogels, scaffolds/patches, sponges, films, and bioactive nanomaterials, tailored to particular wound types and healing phases and able to play a fundamental role in promoting skin regeneration and opening a new era of innovative cosmetic dermatology.^6−8^ Among the biomaterials, natural polymers are preferred to the synthetic ones, tissue engineering, and drug delivery due to their excellent biocompatibility and biodegradability, the reduced risk of inflammation, immunological reaction, as well as endotoxin and virus contamination. They show reduced ethical issues, higher scalability, and environmentally favorable properties.^9^ Furthermore, in various cosmetic products, they can be used as thickeners, film-formers, and conditioning agents.^10,11^ However, finding the optimal combination of compounds (i.e., biomaterials and bioactive agents) is essential to restore the natural mechanisms of endogenous skin that renew skin layers and lessen wrinkle appearance, avoiding the formation of scars and hypertrophic skin. Specialized biopolymers, which are vital for skin regeneration and repair processes, must have the appropriate water absorption ratio to control the moisturizing properties of the skin or hydrate globular proteins to promote their biological activities.^12^ Furthermore, it should be possible for these bioactive chemicals to replicate an environment similar to the extracellular matrix (ECM) seen in nature, where various cells precisely coordinate the molecular and biological processes of cell migration, proliferation, and ECM remodeling.^13^ Finally, a perfect balance between physicochemical properties (e.g., swelling behavior, degradation time), mechanical properties, and antibacterial properties should be also ensured.^1^ One of the polymers that are gaining popularity in pharmaceutical, medical, and cosmetic fields is chitosan (CTS).^12,14^ CTS is a biopolymer derived from chitin, produced through the process of its deacetylation, and it is composed of two units: d-glucosamine and *N*-acetyl-d-glucosamine.^15,16^ In nature, it is found in sea animals, such as lobsters and shrimps, insects (scorpions and spiders), or from microorganisms (green or brown algae and the cell walls of fungi).^16^ Intrinsic properties of CTS, such as molecular weight (*M*_W_) and degree of deacetylation (DDA) largely influence the final properties of chitosan-based skincare products. Furthermore, it holds great potential due to its antioxidant, regenerative, bioadhesive, hemostatic, and antibacterial properties.^17^ Equally important features include biocompatibility, biodegradability, nontoxicity, nonantigenic, and anti-inflammatory activity.^18,19^ Indeed, it might support the healing process by aiding in the histoarchitectural remodeling of skin tissue, macrophage activity, and inflammatory cell activation and proliferation in granular tissues.^20^ Furthermore, CTS can be easily chemically modified to improve its physicochemical properties by blending it with other biomaterials or functionalizing with other (bio)molecules to enhance its biological/antimicrobial activity.^20−23^ For example, Ma et al. employed sorbitol (S) as a plasticizer up to 70% to enhance the physicochemical characteristics of CTS films.^24^ Indeed, as a plasticizer, compared to other polyols, S has demonstrated enhanced mechanical and physicochemical properties.^25^ From a merely technological point of view, CTS can be also easily processed by different routes.^23^ It can be administered in a number of ways due to its inherent mucoadhesive properties and excellent film-forming capabilities.^20^ Among natural compounds, aloe vera (A) has been widely used in wound dressings because of its healing properties, enhancing the bioactivity of CTS.^20,26^ A (*Aloe barbadensis Miller*) is a herbaceous succulent plant that thrives in tropical climates. Already widely known in ancient Egypt and Mesopotamia and in traditional Chinese and Ayurvedic medicine, A has become one of the most frequently used plants for skin care, treatment of various ailments, and aesthetics.^27^ The two primary components of fresh A leaves are a clear mucilaginous gel made from the leaf pulp that is used topically to treat burns and wounds and a bitter yellow juice, primarily containing anthraquinones, regulated by the Food and Drug Administration (FDA) as a laxative and cathartic agent. The colorless mucilaginous gel that is extracted from the parenchymatous cells in young leaves presents two phases: a liquid water-based phase (99–99.5%) and a solid phase (0.5–1.0%) that contains proteins, nonstarch polysaccharides, lignin, vitamins, minerals, and mono- and polysaccharides.^20,28,29^ Polysaccharides and glycoproteins provide the anti-inflammatory properties, while glucomannan is primarily responsible for the healing effect by promoting fibroblast activity and proliferation and increasing the formation of collagen.^30^ Furthermore, A contains antiseptic and antimicrobial properties. Yoshida et al. dispersed A extract in CTS films and evaluated its effect on the films’ properties. Results showed that A was able to enhance the film’s barrier properties, to decrease the films’ absorption capacity, suggesting a cross-linking behavior. The elongation at the break decreased with A addition. Thermal analysis showed that the A increased the stability below 200 °C.^26^ In a more recent study, Genesi et al. created CTS films functionalized with copaiba and A using the casting approach. They then tested the films’ cytotoxicity, antibacterial activity, and *in vivo* healing capability in rat model.^20^ The cytotoxicity of films made with 2% CTS and up to 1% A and copaiba oleoresin in the Balb/c 3 T3 clone A31 cell line exhibited minimal toxicity, while the results indicated that none of the CTS films encouraged microbial permeability. According to *in vivo* data, the 0.5% copaiba-loaded and 0.5% A-loaded CTS films functioned better than the commercial dressings. All studied groups showed a completely developed epithelium, although groups treated with formulations appeared to have more vessel neoformation than those treated with the control.^20^

Our previous research showed also the importance of using d-Glucono-1,5-lactone, known as gluconolactone (GL), as a bioactivated agent of konjac glucomannan films for wound dressing applications. Indeed, the inclusion of GL improved the films’ capacity to stimulate human dermal fibroblasts’ metabolic and energetic activities, encouraging cell division and improving the effectiveness of wound healing in general.^30^ However, to the best of our knowledge, a combination of those components, aloe vera, gluconolactone, and sorbitol in CTS films for wound healing applications, has not been exploited yet.

Herein, the aim of the present research was to investigate the ability of natural polymers, particularly CTS, to develop wound dressing materials, focusing on their film-forming ability and biocompatibility. The study also aimed at highlighting the significance of A as a multifunctional ingredient in skincare products, due to its anti-inflammatory, antibacterial, wound healing, antiaging, skin-protecting, and moisturizing effects. Furthermore, films were functionalized by adding GL or S as a plasticizer.

## Experimental Section

### Chemicals

#### For Film Preparation

Chitosan (CTS, Pol-Aura), aloe vera (A, aloe extract concentrated 10 times, Zrób sobie krem company), delta-glukonolactone (GL, thermo scientific), and d-sorbitol (S, Pol-Aura) were used in the experimental studies.

#### For Physicochemical Studies

Diiodomethane (99%) was purchased from Sigma-Aldrich (Poznań, Poland). Glycerine, sodium hydroxide, and hydrochloric acid for analysis grade were from Avantor Performance Materials Poland S.A. (Gliwice, Poland).

#### For Biological Studies

HaCat cells (Thermo Fisher Scientific, Waltham, MA) were cultured inα-MEM supplemented with 10% fetal bovine serum (FBS, Merck, Darmstadt, Germany) and antibiotics. Cell viability was determined using a CellTiter96Aqueous One Solution Cell Proliferation Assay (MTS, Promega, Poland). A lactate dehydrogenase (LDH) assay was conducted using a Cytotoxicity Detection Kit (Roche, Germany). The amount of nitric oxide (NO) was detected by using a colorimetric Griess reaction. Sodium hydroxide (Alchem, Toru, Poland), fluorescein diacetate (Sigma-Aldrich, Pozna, Poland), Tryptone Soya Broth (TSB), (Pol-Aura, Poland) phosphoric acid (Sigma-Aldrich, St. Louis, MO), and Plate Count Agar (PCA, Biomaxima, Lublin, Poland) were used for antibacterial studies.

### Chitosan Characterization

The CTS moisture content was determined gravimetrically by drying a known mass of CTS under reduced pressure at 40 °C to a constant weight. The moisture content was defined as the mass of water per mass of the CTS calculated as the mean of five independent measurements.

The CTS deacetylation degree (DD) was characterized by using potentiometric titration. The details of the measurement methodology have been given by us earlier.^31^ A Potentiometric Microtitrator (Cerko Lab System, Gdynia, Poland) was used throughout the study. DD was calculated according to the formula as mean result of three independent titrations:
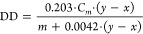
where:

*m* is the mass of the CTS [g], *x* is the volume of NaOH solution used on the HCl titration (first equivalence point) [cm^3^], *y* is the volume of NaOH solution used on titration of both HCl and amine groups of CTS (second equivalence point) [cm^3^], and *C*_*m*_ is the molar concentration of NaOH solution [mol dm^–3^].

### Materials Preparation

CTS was dissolved at a concentration of 1% in 0.1 M acetic acid. The CTS solution was mixed with a solution of 10-fold concentrated aloe (2 and 5 w/w%) and separately with the addition of solid S (1 w/w%) or solid GL (1 w/w%). The weight ratios of the above mixtures are given in Table 1. The solutions were stirred with a magnetic stirrer for 1 h and then poured into a plastic holder (40 mL; 10 cm × 10 cm). Thin films were produced by evaporating the solvent at room conditions (thickness 0.13 ± 0.02 μm). Pure CTS-based films, as well as CTS films containing GL and S, were utilized as controls for comparison (Table 1).

**Table 1:** Nomenclature and Chemical Composition of the Different Films

abbreviation	sample
100CTS	film based on chitosan
98CTS/2A	film based on chitosan with the 2 w/w % content of aloe vera
95CTS/5A	film based on chitosan with the 5 w/w % content of aloe vera
99CTS/S	film based on chitosan with the 1 w/w % content of sorbitol
97CTS/2A/S	film based on chitosan with the 2 w/w % content of aloe vera and 1 w/w % content of sorbitol
94CTS/5A/S	film based on chitosan with the 5 w/w % content of aloe vera and 1 w/w % content of sorbitol
99CTS/GL	film based on chitosan with the 1 w/w % content of gluconolactone
97CTS/2A/GL	film based on chitosan with the 2 w/w % content of aloe vera and 1 w/w % content of gluconolactone
94CTS/5A/GL	film based on chitosan with the 5 w/w % content of aloe vera and 1 w/w % content of gluconolactone

### Materials Characterization

#### Attenuated Total Reflection-Fourier Transform Infrared (ATR-FTIR)

A Nicolet iS5 spectrometer (Thermo Fisher Scientific, Waltham, MA) equipped with ID7 ATR and a ZnSe crystal was used to capture the films’ infrared spectra in the range of 4000–550 cm^–1^, at room temperature, in an air atmosphere. The resolution was 4 cm^–1^. An average of 32 scans was considered.

#### Atomic Force Microscopy (AFM)

Surface roughness was analyzed using a NanoScope IIIa MultiMode Scanning Probe Microscope (Veeco Metrology, Inc., Santa Barbara, CA) The selected parameters were tapping mode; room temperature; and an air atmosphere. The root-mean-square (*R*_q_) roughness and the arithmetic mean roughness (*R*_a_) were calculated using Nanoscope Analysis v6.11 software (Bruker Optoc GmbH, Ettlingen, Germany).

#### Water Content

The water content of the films was determined by using a gravimetric approach based on the oven-drying method. Initially, samples were weighed and then dried in an oven set at 105 °C, until a constant weight was achieved, ensuring the complete removal of moisture. The water content was then calculated and expressed as the amount of water (in grams) per 100 g of the dry sample. The measurements were performed in quintuplicate (*n* = 5) to ensure accuracy and reproducibility.

#### Surface Free Energy

Surface free energy was assessed using the Owens-Wendt method. That allows the formation of noncovalent contacts between the liquid and the film surface.^32^ Surface free energy-IFT (s), along with its polar-IFT (s,P) and dispersive-IFT (s,D) components, can be determined through contact angle measurements. A goniometer with a drop shape analysis system (DSA 10 Control Unit, Krüss, Germany) was used to measure the contact angles of glycerin and diiodomethane at a constant temperature.

#### Mechanical Properties

Mechanical properties were evaluated by using a Shimadzu EZ-Test apparatus (EZ-SX, Kyoto, Japan). Samples of known thicknesses were placed between grips and stretched at a speed of 5 mm/min. The Young’s modulus was determined from the linear region of the stress–strain curve using the Trapezium × Texture software. Each test was performed 10 times for accuracy.

#### Biocidal Activity

The biocidal properties of the tested films were evaluated in accordance with the ISO 22196:2011 standard.^33^ Biocidal activity was tested against pathogens *Pseudomonas aeruginosa* ATCC 15442, *Staphylococcus aureus* ATCC 6538, and *Escherichia coli* ATCC 8739. The bacterial strains were cultured in TSB at 37 °C for 24 h. Subsequently, 2 mL of the suspension was centrifuged at 10,000 rpm using a MiniSpin centrifuge (Eppendorf). The pellet was resuspended in 1 mL of sterile saline, and the optical density was adjusted using a densitometer (Densi-La-Meter II, Erba Lachema, Czech Republic) to OD = 0.5, corresponding to 1.5 × 10^8^ bacterial cells per 1 mL, according to the McFarland scale.

Next, an experimental setup was prepared loosely based on the method described by Richert et al.^34^ It consisted of a Petri dish containing a 2.5 cm × 2.5 cm sample of the tested material, sterilized with UV light for 20 min on each side. 100CTS films were treated as a control sample. A 0.1 mL bacterial suspension was applied to the film and covered with a sterile 2 cm × 2 cm parafilm to prevent evaporation and ensure even distribution of microorganisms on the film surface. A piece of sterile filter paper soaked in 2 mL of sterile distilled water was placed in the dish, making sure it did not come into contact with the film, to keep the atmosphere humid. For a whole day, the prepared plates were incubated at 37 °C. The quantity of bacterial cells on the tested and control materials was counted following incubation. To recover the bacteria from the surface of the materials, the film sample along with the parafilm was placed in 10 mL of neutralizer medium and shaken for 3 min. Following the preparation of a series of dilutions, the bacteria were inoculated, using the pour plate method on PCA medium with the composition [g/L]: yeast extract, 2.5; tryptone, 5.0; glucose, 1.0; agar, 15.0; distilled water—1 L, pH 7.0–7.2, and incubated for 24 h at 37 °C. After incubation, the bacteria were counted, and the antibacterial activity was calculated according to the ISO standard based on the average logarithmic numbers of viable bacteria at time = 0 and 24 h. According to the standard, an *R*-value (Reduction of microorganism count) of 2 or greater indicates the antimicrobial activity of the tested material.

#### Biodegradation of Materials in Soil and Enzymatic Activity

Biodegradation of films was assessed by measuring the biochemical oxygen (O_2_) demand (BOD) with the OxiTop Control (WTW, Wrocław, Poland) according to the operating instruction provided by the supplier WWT (1998) and the method described by Swiontek Brzezinska et al.^35^ 100 g of soil was placed in the OxiTop measuring dishes along with 0.5 g of test films. The measuring heads were affixed, and the quivers with the CO_2_ absorbent (0.4 g NaOH) were put inside the measuring dishes. The incubation period lasted 21 days at 20 °C. A soil-only test, with no film added, was treated as a control.

From the soil after biodegradation of the test materials, 10 g was measured and dissolved in 90 mL of sterile saline; 10 mL was measured, and 0.1 mL of 1 mg/mL fluorescein diacetate (Sigma-Aldrich, Pozna, Poland) was added. A sample without the addition of fluorescein diacetate was prepared as a control. The samples were incubated for 30 min at 30 °C without light. After incubation, 2 mL were transferred to Eppendorf-type tubes, and centrifuged at 10,000 rpm for 5 min. The Hitachi F 2500 spectrofluorometer (Thermo Fisher Scientific, Waltham) was used to measure the intensity of the fluorescence at 480 nm for excitation and 505 nm for emission. The result was determined as μg of fluorescein per 1 g of soil dry weight.

#### Cytocompatibility

Cell culture analyses included two experimental setups. In the first system, in order to check the effect of direct contact of cells with the material, HaCat cells were cultured in α-MEM supplemented with 10% FBS and antibiotics on the surface of the material in density 2 × 10^4^cells/cm^2^ for a period of 72 h. The second set of experiments was aimed at checking the effect of substances released from the surface of the material on the behavior of the cells. For this purpose, the cells were cultured at the same density on culture plastic, and for a period of 24 h, they were exposed to extracts obtained as a result of 72 h of incubation of materials in the culture medium itself. In both systems, the cells were cultured in 24-well plates.^36^ To assess the cell viability as previously described,^37−39^ following a PBS wash, each well received 0.2 mL of a 10% 3-(4,5-dimethylthiazol-2-yl)-5-(3-carboxymethoxyphenyl)-2-(4-sulfophenyl)-2H-tetrazolium (MTS) reagent solution in phenol-free α-MEM. At 37 °C, the plates were incubated until they appeared to turn from yellow to brownish. After that, the media were moved to 96-well plates, and a plate reader was used to measure the absorbance at 492 nm. Following the technique, a colorimetric Griess reaction was used to determine the quantity of NO. Griess A (0.1% *N*-1-naphthylethylenediamine dihydrochloride), Griess B (1% sulfanilamide in 5% phosphoric acid), and an equivalent volume of the collected samples (50 μL) were combined on a 96-well plate. The cultures were washed with PBS and 0.2 mL of a solution of 10% MTS reagent. The intensity of the formed color was measured at a wavelength of 540 nm. An LDH assay was conducted as previously described.^40^ Briefly, after gathering the culture media, 50 μL of each sample was put into a 96-well plate. The samples were then combined with an equivalent volume of reagent combination made in accordance with the manufacturer’s protocol. The intensity of the red color produced in the colorimetric assay was measured at a wavelength of 490 nm following incubation at 37 °C.

### Statistical Analysis

The data was statistically analyzed using SigmaPlot 14 software (Systat Software, San Jose, CA). All of the results were expressed as mean ± standard deviations (SD) and evaluated by one-way analysis of variance (ANOVA). To check for normal distribution, the Shapiro-Wilk test was employed. For multiple comparisons with the control group, the Bonferroni *t*-test was applied, with the statistical significance defined as *p*.

## Results and Discussion

CTS was preliminarily characterized. Results from gravimetric tests showed that the CTS moisture content was 5.42 ± 0.13%. Furthermore, the DD measured by potentiometric titration was 93.8 ± 2.2%.

**Figure 1:**
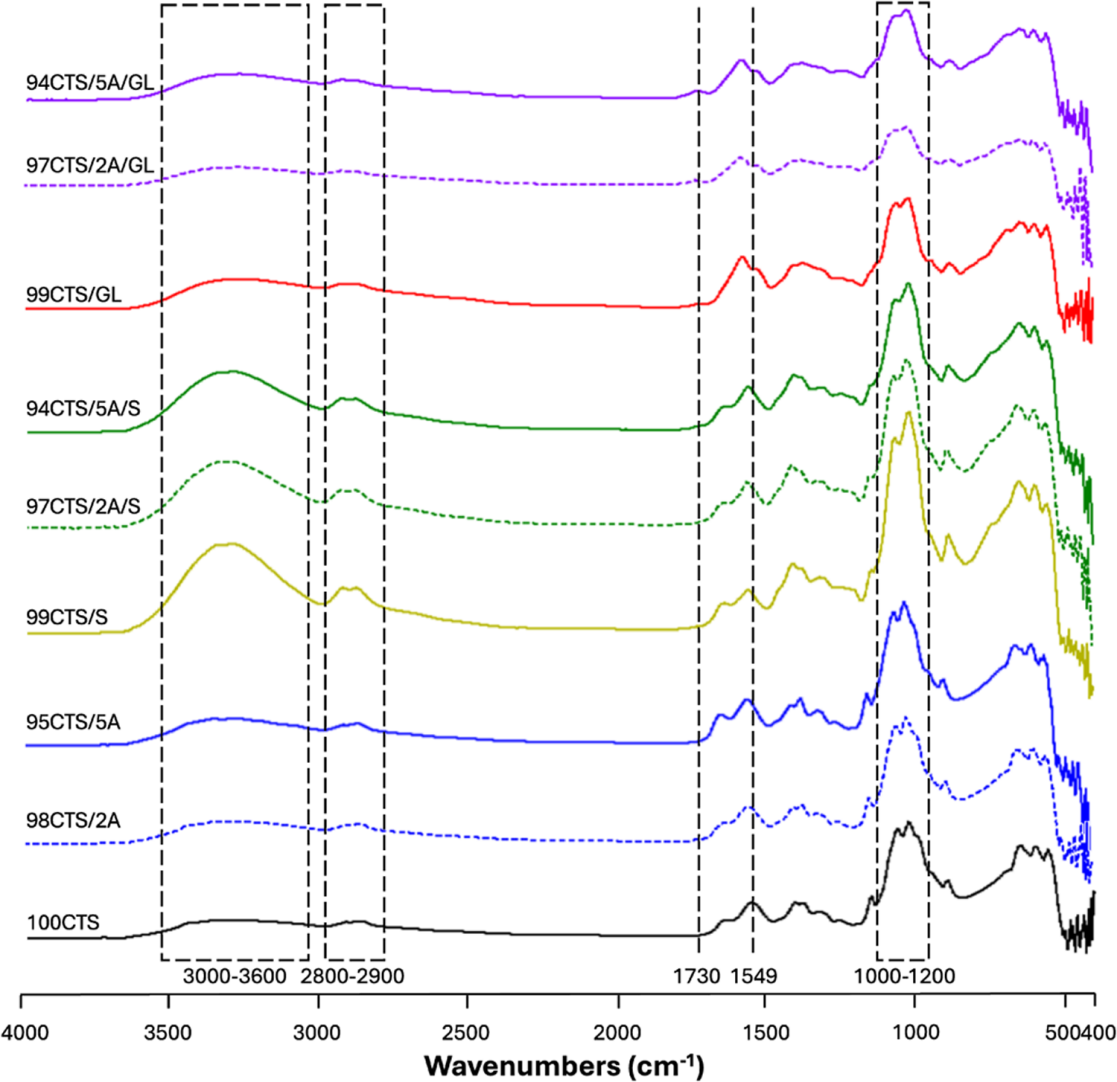
FTIR-ATR spectra of CTS-based films between 4000 and 400 cm^–1^.

In order to exploit the interaction between CTS, S, and GL molecules, bioactivated with different concentrations of A, FTIR experiments were carried out (Figure 1). As it is possible to observe, all of the spectra highlighted the typical broad absorption band between 3600 and 3000 cm^–1^ representing the stretching of O–H groups, ascribable to CTS spectrum (black curve).^23,30^ Furthermore, they showed the absorption bands at ∼1549 cm^–1^ (–NH_2_), ∼1646 cm^–1^ (amide I band), and ∼1318 cm^–1^ (N-acetyl group) characteristic of the polysaccharide structure.^24^ Finally, between 1000 and 1200 cm^–1^, spectra showed the C–O stretching vibrations of the glycosidic linkage.

When S was added, the band between 3600 and 3000 cm^–1^ gradually narrowed and slightly shifted (99CTS/S, 97CTS/2A/S, and 94CTS/5A/S-yellow, dashed green, and solid green curves, respectively). This result proved that the S addition disrupted the hydrogen bonds between CTS molecules. In addition, the absorption band at 1549 cm^–1^ for neat CTS shifted to 1559 cm^–1^ for the composite films, highlighting that the electrostatic interactions in CTS films were weakened by S addition, in agreement with other works reported in the literature.^24^

Looking at the CTS/GL curves (99CTS/GL, 97CTS/2A/GL, 94CTS/5A/GL, reported as red, dashed purple, and solid purple curves, respectively), the characteristic carbonyl band of d-gluconolactone at ∼1730 cm^–1^ can be detected. Furthermore, the same range between 1000 and 1200 cm^–1^ was observed due to the presence of both phenolic hydroxyl and carboxylate groups of GL.^30^

### Atomic Force Microscopy

The AFM results demonstrated that the surface roughness varies significantly depending on the type and concentration of the additives used (Table 2). The pure chitosan (100CTS) film exhibited a moderate level of roughness, indicating a relatively smooth surface structure typical for unmodified CTS, with few peaks or valleys. The addition of A had a considerable impact on the surface texture. Films containing 5% aloe (95CTS/5A) showed the highest roughness among all samples with elevated *R*_q_ and *R*_a_ values, indicating a significantly more textured surface. This increased roughness could result from the interaction between CTS and A, disrupting its uniform network and creating microstructural features that contribute to a rougher surface. In contrast, films with 2% aloe (98CTS/2A) displayed lower roughness levels, suggesting that higher A concentrations lead to more extensive surface modification, potentially due to increased molecular interactions with the matrix. Our results agree with previous works reported in the literature. For example, Janczak et al. designed films composed of potato starch, CTS, and A gel and they demonstrated that the increase in A content enhanced the roughness of the material surface.^41^ Sharma et al. recently discovered a similar trend with polymer hydrogels based on A and sterculia gum intended for wound dressing applications.^42^

**Table 2:** Roughness Parameters and Water Content Values for All Studied Films (*n* = 5; * Significantly Different from 100CTS—*p* < 0.05)

specimen	*R*_a_ [nm]	*R*_q_ [nm]	water content [g/100]
100CTS	1.50 ± 0.17	1.89 ± 0.18	6.87 ± 1.84
98CTS/2A	0.19 ± 0.04*	0.26 ± 0.07*	28.61 ± 3.39*
95CTS/5A	5.37 ± 0.09*	7.91 ± 0.11*	23.09 ± 1.87*
99CTS/S	0.21 ± 0.03*	0.30 ± 0.04*	21.72 ± 3.70*
97CTS/2A/S	0.32 ± 0.05*	0.52 ± 0.15*	17.69 ± 2.59*
94CTS/5A/S	0.20 ± 0.05*	0.26 ± 0.12*	23.26 ± 1.15*
99CTS/GL	0.22 ± 0.03*	0.28 ± 0.11*	14.59 ± 1.76*
97CTS/2A/GL	1.00 ± 0.19*	1.27 ± 0.22*	11.04 ± 2.49
94CTS/5A/GL	1.40 ± 0.11	1.75 ± 0.08	12.29 ± 2.25

Films containing S and GL exhibited distinct surface characteristics. Formulations with sorbitol, such as 99CTS/S, showed reduced surface roughness compared to aloe-only films, indicating that S might act as a plasticizer, enhancing film smoothness and minimizing surface irregularities. Similarly, gluconolactone-containing films (e.g., 99CTS/GL) demonstrated low roughness values, suggesting that GL contributes to a smoother surface by potentially enhancing the homogeneity of the film structure.

Formulations with combined additives, such as aloe and gluconolactone (e.g., 94CTS/5A/GL), displayed moderate roughness values. This balanced roughness may reflect an interplay between the roughness-inducing effect of A and the smoothing influence of GL. Similarly, films with both aloe and sorbitol (97CTS/2A/S) showed a lower level of roughness, indicating that these additives can be combined to fine-tune the surface texture.

### Water Content

The incorporation of additives, such as A, GL, and S into the CTS matrix significantly influenced the water content of the films (Table 2). Pure CTS films exhibited the lowest moisture retention, highlighting their limited hydrophilic capacity in the absence of modifying agents. The addition of A markedly increased the water content, reflecting its hydrophilic properties,^43^ which facilitate water retention by introducing additional hydrogen bonding sites within the matrix. Similarly, S, known for its hygroscopic nature,^44^ enhanced water absorption, although its effect was less pronounced compared to A. The inclusion of GL also resulted in increased water content, though to a lesser extent, likely due to its moderate ability to interact with water molecules.^45^ Combinations of additives further altered the films’ water retention behavior. Mixtures of A and S or A and GL generally produced intermediate water content values, demonstrating that the specific interplay between components can modulate the hydrophilicity and moisture-holding capacity of the films.

These findings underscore the importance of additive type and concentration in tailoring the water content of CTS-based films. Hydrophilic additives such as A and S disrupt the tight CTS structure, increasing porosity and creating additional sites for water interactions. This structural modification enables greater moisture retention, which can be leveraged to optimize the material’s properties for specific applications, such as wound healing. Conversely, films with a lower water content may be preferred for applications requiring greater stability and reduced susceptibility to moisture. Chelu et al.^100,65^ studied the water content properties of films based on xanthan gum containing A. The results showed that increasing A concentration led to a corresponding increase in moisture content. Similar results were observed in our study of films containing plasticizers.

### Surface Free Energy

The surface free energy was evaluated by measuring the contact angle of both polar and nonpolar liquids placed on the film surface (Table 3). The values of surface free energy are crucial, as they determine the presence of dangling bonds, which in turn regulate interactions between cells and materials. Higher surface free energies can inhibit these interactions. The results of the study of the interfacial surface tension of CTS films with various additives demonstrate that the film composition exerts a significant influence on the measured values of this parameter. Pure CTS films exhibited higher surface tension, which was altered with the introduction of additives such as A, S, and GL. Bajer et al.^41^ obtained similar results. They studied the surface free energy of films based on starch/CTS mixture containing A. The results showed that the addition of A into biopolymer films did not cause a significant change in calculated free surface energy.

**Table 3:** Surface Free Energy (IFT (s)), Its Polar (IFT (s,P)), and Dispersive (IFT (s,D)) Components of Films Based on Chitosan (*n* = 5; * Significantly Different from 100CTS)

specimen	IFT (s) [mJ/m^2^]	IFT (s,P) [mJ/m^2^]	IFT (s,D) [mJ/m^2^]
100CTS	35.59 ± 1.01	8.79 ± 0.12	26.80 ± 0.22
98CTS/2A	34.72 ± 0.77	8.64 ± 0.28	26.08 ± 0.49
95CTS/5A	34.48 ± 1.13	10.61 ± 0.44*	23.87 ± 0.69*
99CTS/S	33.10 ± 1.74*	10.73 ± 0.88*	22.37 ± 0.86*
97CTS/2A/S	32.70 ± 0.41*	10.53 ± 0.17*	22.17 ± 0.24*
94CTS/5A/S	36.12 ± 0.52	13.53 ± 0.22*	22.59 ± 0.30*
99CTS/GL	33.09 ± 1.73*	10.72 ± 0.87*	22.37 ± 0.86*
97CTS/2A/GL	32.78 ± 0.93*	15.76 ± 0.58*	17.02 ± 0.35*
94CTS/5A/GL	34.64 ± 1.28	14.96 ± 0.53*	19.68 ± 0.75*

An increase in A content within the CTS films led to a general moderate reduction in surface tension, affecting both the overall tension and its components related to interactions with polar and nonpolar phases. The incorporation of S into the film composition further modified these values, resulting in a continued decrease in surface tension. The most pronounced changes were observed when larger quantities of A and S were simultaneously introduced.

Conversely, the addition of GL induced significant alterations in interfacial surface tension, particularly with regard to the polar phase, where an increase in this component’s concentration resulted in a marked rise in IFT (s,P), which can be due to the hydrophilic nature of GL.^46^ In contrast, for the nonpolar phase (IFT (s,D)), the trend was reversed; this parameter decreased with the addition of GL, suggesting strong interactions between this component and the film surface.

### Mechanical Properties

**Figure 2:**
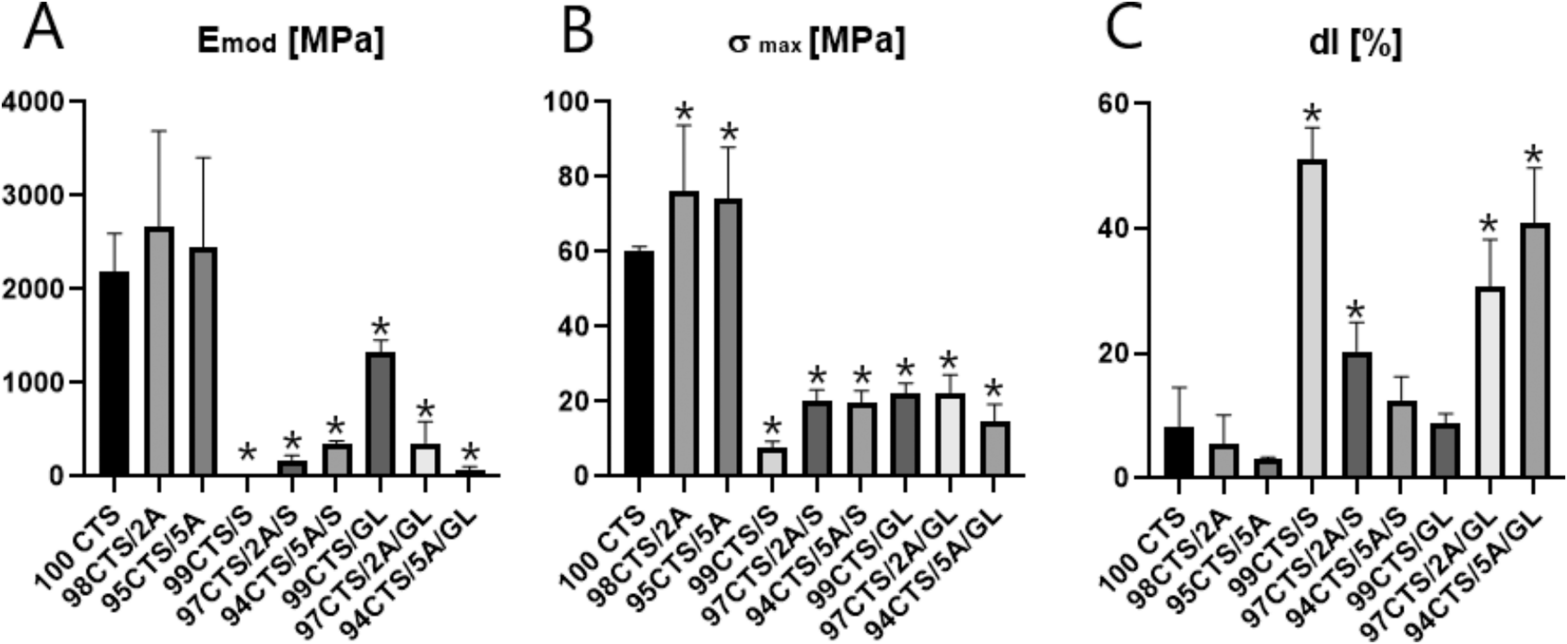
(A) Young’s modulus (*E*_mod_), (B) maximum tensile strength (σ_max_), and (C) elongation at break (d*l*) determined for the different CTS-based films. (*n* = 10; significant differences from 100CTS (*p* < 0.05)).

The mechanical behavior of CTS films with various additives was evaluated in terms of Young’s modulus (*E*_mod_), maximum tensile strength (σ_max_), and elongation at break (d*l*) (Figure 2). *E*_mod_ for pure chitosan (100CTS) exhibited a relatively high value, which gradually decreased with the introduction of additives. Samples containing 2% aloe (98CTS/2A) and 5% aloe (95CTS/5A) also maintained high modulus values; however, the addition of larger quantities of S and GL significantly reduced this parameter. The greatest reduction in stiffness was observed in the samples with 5% aloe and sorbitol (94CTS/5A/S), indicating increased material flexibility.

σ_max_ also varied, depending on the film composition. Pure CTS exhibited one of the highest tensile strength values, and the introduction of A did not cause a decline in strength. However, the inclusion of GL and S combined or not with A, reduced the maximum tensile strength, suggesting that these additives weaken the mechanical strength of the material. (d*l* for pure CTS films was one of lowest among all tested samples. Increasing the A content alone did not significantly enhance the material’s ability to elongate. Similarly, when GL was added to CTS, d*l* did not change. However, when A was combined with GL, d*l* values increased, particularly in the sample with 5% A. The addition of S further increased the film’s elasticity; meanwhile, the elongation values decreased when S was coupled with A.

A wide range of mechanical properties have been documented in earlier research on creating CTS-based scaffolds for skin tissue engineering applications. For instance, to enhance the healing process of wounds, Nokoorani et al.^22^ developed scaffolds based on CTS and gelatin that contained 0.25, 0.5, 0.75, and 1% allantoin. The specimens’ Young’s modulus ranged from 0.0654 ± 0.0207 to 0.1291 ± 0.0355 MPa, their elongation at break ranged from 81.17 ± 29.96% to 109.98 ± 42.14%, and their tensile strength ranged from 0.0406 ± 0.0030 MPa to 0.0992 ± 0.0230 MPa. The results show that the specimen with 0.5% allantoin had the most rigid structure (*E*_mod_ = 0.1291 ± 0.0355 MPa), whereas the sample with 1% allantoin had the most flexible structure (*E*_mod_ = 0.0654 ± 0.0207 MPa). Our findings are consistent with several investigations in the literature, despite the difficulty in comparing the mechanical properties of films because of variations in parameters including porosity, pore size, pore interconnectivity, and the procedures used to fabricate and cross-link the samples.^47−49^ Nonetheless, it is worth noting that given the particular application, the obtained Young’s modulus values are within the range of those determined by Agache et al. (0.42 MPa for young people and 0.85 MPa for the elderly), indicating its possible application as a material for wound dressings.^50^

### Biocidal Properties of Materials

**Figure 3:**
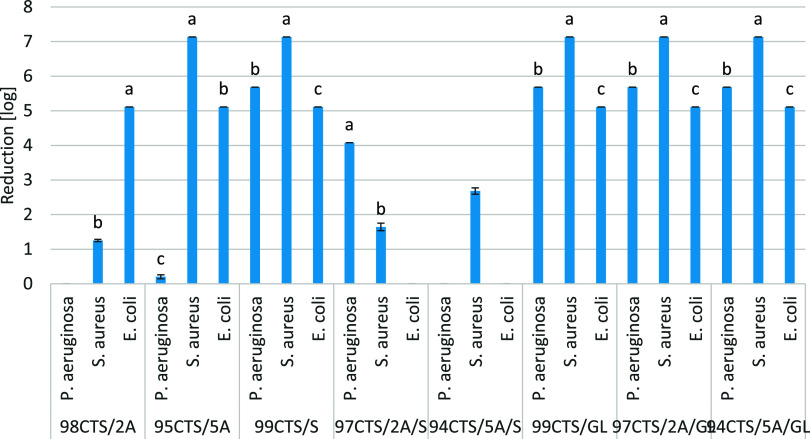
Antimicrobial activity of films against selected pathogens. Statistical significance was tested within a film type. Letters on the bars of the figure indicate statistically significant differences (*p* < 0.05). Different letters indicate statistically significant results, and the same letters indicate no statistical significance.

The biocidal activity of the tested materials was calculated considering the control sample, i.e., 100CTS at *t* = 0 and after 24 h (Figure 3). The films 99CTS/S, 99CTS/GL, 97CTS/2A/GL, and 94CTS/5A/GL were effective against the three tested pathogens. The remaining materials exhibited biocidal effects against two or more of the tested pathogens. The results indicate that very good biocidal properties were achieved by materials with the addition of GL, S, or a combination of GL and A. The addition of A alone, or A with S, does not demonstrate a satisfactory level of biocidal activity against all pathogens.

Studies have shown that the polysaccharides and glycoproteins present in the pulp of A leaves have medicinal properties.^51^ In addition, A extract contains antiseptic and antimicrobial properties ascribable to lupeol, salicylic acid, urea nitrogen, cinnamic acid, phenols, and sulfur, which exhibit inhibitory action against viruses, bacteria, and fungi.^52,53^ Enzymes (such as amylase and lipase) present in its extract can aid digestion by breaking down fats and sugars, and carboxypeptidase inactivates bradykinins and produces anti-inflammatory effects.^54^ Our studies show that aloe vera alone incorporated into CTS did not have biocidal properties against the pathogens tested. Only the combination of CTS containing A and GL showed antimicrobial activity. At the same time, CTS containing only S or GL showed a high reduction in the abundance of strains tested. Genesi et al.^20^ developed CTS films containing A and copaiba oil, both as potential wound healing materials. For comparison, they used CTS containing silver sulfadiazine. Their research showed that A did not exert antimicrobial activity against *E. coli*, *S. aureus*, and *P. aeruginosa* even at the highest concentrations of these bioactive compounds in CTS films except films with silver sulfadiazine. However, the authors confirmed that the developed films accelerated wound healing. In contrast, Szadkowski et al.^55^ reported that coatings based on CTS with essential oils and plant extracts (i.e., sage, lavender, and A) increased biocidal activity against *E. coli*, *Bacillus subtilis*, *S. aureus*, *Candida albicans*, and *Aspergillus niger*. The most promising results were obtained for the coating containing CTS, A, and cinnamon essential oil. Nwe et al.^56^ prepared modified CTS-alginate-starch-S composite membranes (CASS) for biomedical applications. According to the authors, CASS (0.1% S) composite membranes showed antimicrobial activities against *B. subtilis*, *S. aureus*, *P. aeruginosa*, *Bacillus pumilus*, *C. albicans*, and *E. coli*. Antimicrobial activity is highly dependent on the type and concentration of the biocidal substance. As individual compounds, they can have a strong biocidal effect or a weaker effect when incorporated into the polymer. Sorbitol and gluconolactone are used in the production of cosmetics to extend shelf life as natural preservatives. When these compounds are combined with polymers, they can exhibit synergistic effects and increase the efficacy. Our research suggests that CTS may exhibit antimicrobial activity^57^ and S and GL may support the action and reduce water loss.^58^

### Biodegradation of Materials

**Figure 4:**
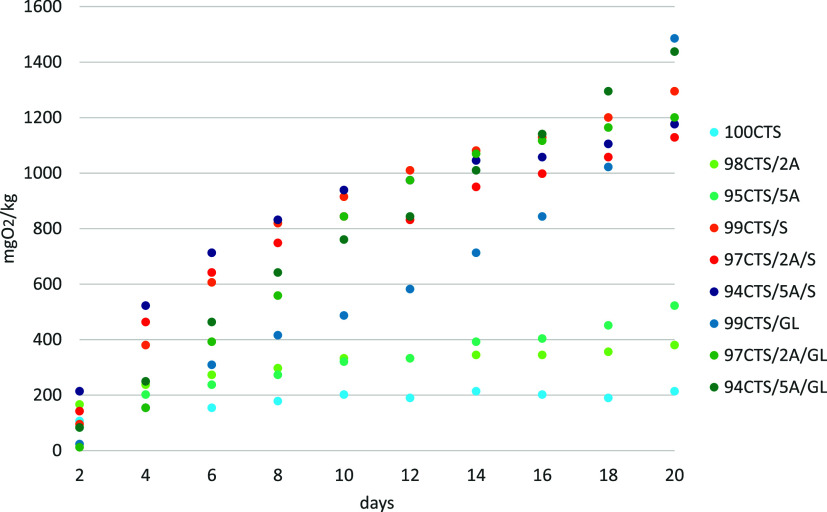
Biodegradation of materials in soil.

The biodegradation results of the tested materials are shown in Figure 4. The results were reduced by the respiratory activity in the control sample, i.e., in soil alone, without the addition of films. The lowest O_2_ consumption by soil microorganisms was for the 100CTS sample, at 213.88 mg O_2_/kg after 20 days. All other films that were modified by the addition of A, S, or GL showed higher BOD values. The highest O_2_ consumption after 20 days was in the sample containing the 99CTS/GL film, at 1485.28 mg O_2_/kg. The second highest result was in the sample with 94CTS/5A/GL film and was 1437.75 mg O_2_/kg.

**Figure 5:**
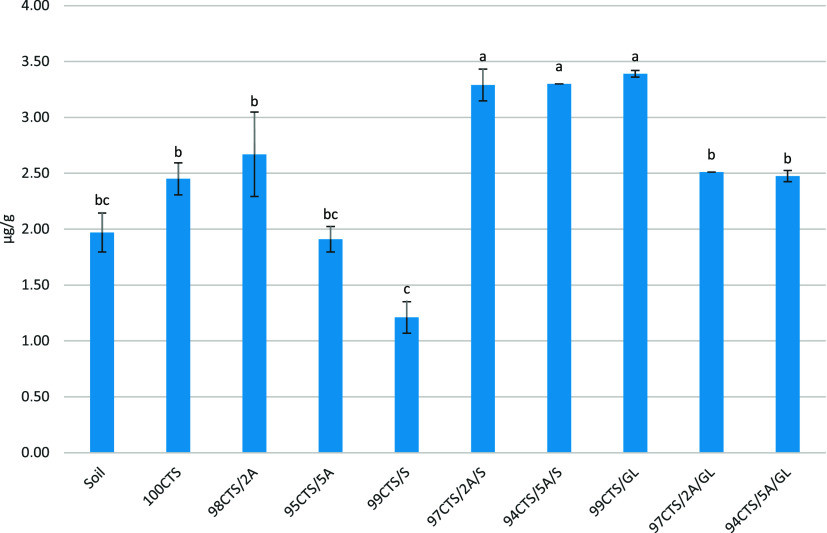
Effect of films on changes in soil hydrolytic enzyme activity. Letters on the bars of the figure indicate statistically significant differences (*p* < 0.05). Different letters indicate statistically significant results, and the same letters indicate no statistical significance.

Soil microorganisms secrete extracellular enzymes that play an important role in the decomposition of organic matter but are also indicators of soil quality.^59,60^ The results shown in Figure 5 indicate that soil microorganisms after contact with the tested materials did not reduce the activity of hydrolases and, thus, did not interfere with the degradation processes in soil. The 94CTS/5A/S and 99CTS/GL films significantly increased hydrolase activity.

Determining the degree of biodegradation of materials with antimicrobial properties is an important parameter affecting the biology of the soil environment. Our study showed that pure CTS was the most difficult to degrade. However, chitosan films modified with S and GL provided the best carbon source for microorganisms. Das et al.^61^ and Nakkabi et al.^62^ formulated a CTS film modified with chlorella. The authors found that films’ original appearance and structural integrity were lost and that their surfaces were rougher and more degraded, with holes and pits that were porous, indicating a significant degree of biodegradation by the soil’s microorganisms. Similar results were obtained by Deshmukh et al.^63^ Trang et al.^64^ prepared and characterized a CTS film modified with A at concentrations of 5, 10, and 15%. The authors confirmed that the presence of A promoted biodegradation. Our results confirm that the addition of anti-inflammatory compounds to CTS films can promote biodegradation. Microorganisms use more of the oxidized aqueous O_2_ to oxidize films, indicating faster biodegradation. At the same time, the hydrolases we tested were also active and their activity was not affected by the presence of CTS films.

### Cytocompatibility

**Figure 6:**
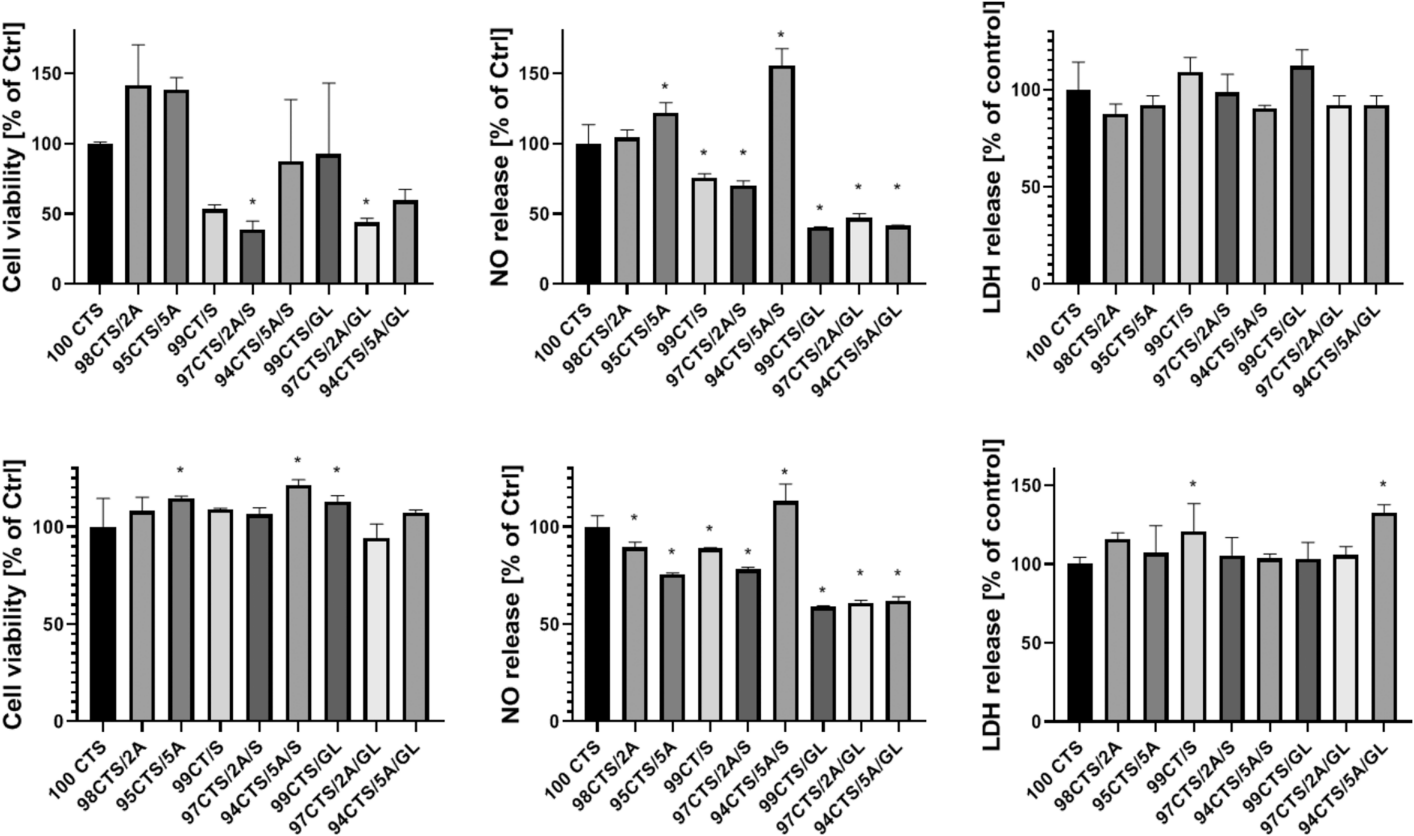
Cell viability and release of NO and LDH. Cells cultured directly on the surface of the material, bottom row: Cells cultured in the presence of material extracts (*n* = 3; * significantly different from 100CTS—*p* < 0.05).

Viability and cytotoxicity tests are one of the basic procedures to assess the biocompatibility of a material *in vitro*.^65^ It is also worth mentioning that in the case of skin, the synthesis of NO is very important. It plays a key role in the skin’s response to external stimuli such as ultraviolet (UV) mesh, wound healing, or reaction to infection.^66^ In the case of exposure of cells to material extracts, no large effect of reduced cell viability was observed, which in most cases corresponded to cytotoxicity (Figure 6). The only extract that stimulated NO synthesis by cells was an extract obtained from the 94CTS/5A/S material which was also observed in cells cultured on the material. Contact of cells with the material did not cause a cytotoxic effect, but the materials 97CTS/2A/S and 97CTS/2A/GL inhibit proliferation. Results obtained during cell studies confirm the usefulness of materials containing A.^67^ It is worth mentioning that the dressing material should have its biological effect by releasing the substance.^68^ In the case of the materials studied, the effect of the indirect contact of the released substances did not have a cytotoxic effect and, in some cases, even stimulated cell proliferation which is associated with increasing the content of aloe vera in the matrix.

For potential wound dressing materials, both direct and indirect contact significantly influences the tissue response. If a material is intended to act as a scaffold for diseased tissue, then it should promote cell adhesion and proliferation. Conversely, materials designed as carriers of biologically active substances should not induce cytotoxic effects but rather exert a desired therapeutic response upon the controlled release of bioactive compounds. Additionally, an ideal dressing material should not form a permanent bond with the skin, as its removal could result in mechanical damage to the underlying tissue.^69^ In direct contact conditions, a trend was observed where increasing concentrations and types of bioactive substances (A, S, and GL) incorporated into the matrix led to a reduction in cellular metabolic activity (MTS assay). This effect can be interpreted as a decrease in cell proliferation and adhesion rates. However, despite the lower number of adherent cells, no cytotoxic effects were detected (LDH assay). This reduction in adhesion may also be attributed to surface topography, which could hinder cell colonization compared to the more uniform and flatter surface of standard culture plastics.^70^ Moreover, the increase in the bioactive substance content correlated with a rise in surface energy, which may further influence cell-material interactions. However, in indirect contact conditions, where cells are exposed to substances released from the materials, the topography factor is minimized. Under these conditions, no significant cytotoxicity or proliferation inhibition was observed. Interestingly, some surfaces even slightly stimulated cell growth, suggesting a potential bioactive effect. These findings suggest that at the in vitro level, the tested materials do not exhibit cytotoxicity and are suitable as biocompatible carriers for therapeutic bioactive compounds such as A, S, and GL.

## Conclusions

This study successfully developed and characterized CTS-based films functionalized with A, GL, and S for advanced wound healing applications. The combination of these additives demonstrated synergistic effects, enhancing the films’ physicochemical, mechanical, and biological properties. Aloe vera contributed to improved hydrophilicity and healing properties, while gluconolactone and sorbitol significantly enhanced antimicrobial activity, thermal stability, and flexibility. The biodegradation analysis confirmed that these films provide an eco-friendly solution with accelerated degradation in soil, making them suitable for sustainable biomedical applications. Furthermore, cytocompatibility assessments indicated minimal cytotoxic effects and improved cellular responses, highlighting the potential of these films as biocompatible wound dressings. To find the best compromise among the given properties for CTS films, a balance across all of the properties should be taken into consideration. A summary of the provided data is reported in Table 4.

**Table 4:** Summary of the Main Properties of the Developed Films as Wound Dressings^a^

			surface free energy			mechanical behavior					
abbreviation	roughness	water content	IFT (s)	IFT (s,P)	IFT (s,D)	Young’s modulus	maximum tensile strength	elongation at break	biocidal properties	biodegradation	biocompatibility and NO synthesis
100CTS	++	+	+++	+	+++	+	++	+	+	+	++
98CTS/2A	+	++	++	+	+++	+	++	+	++	++	++
95CTS/5A	+++	+++	++	++	++	+	++	+	+	++	++
99CTS/S	+	+++	+	++	++	+++	+++	+++	+++	++	++
97CTS/2A/S	+	++	+	++	++	+++	+++	++	+	++	+
94CTS/5A/S	+	+++	++	+++	++	+++	+++	+	+	++	+++
99CTS/GL	+	++	+	++	++	++	+++	+	+++	+++	++
97CTS/2A/GL	++	++	+	+++	+	+++	+++	+++	+++	+	+
94CTS/5A/GL	++	++	++	+++	+	+++	+++	+++	+++	+++	++

a Considering the specific feature (i.e., roughness, water content, etc.), the level of adequacy is indicated with + (low), ++ (moderate), and +++ (high). Level of adequacy. + low; ++ moderate; +++ high.

Based on Table 4, the best compromise would be a sample that maintains a moderate to high level of adequacy across most properties, considering wound healing application. In particular, 97CTS/2A/GL and 94CTS/5A/GL dressings showed a high level of adequacy in terms of surface free energy, demonstrating biocidal properties, and adequate biodegradation, with moderately to highly appropriate mechanical behavior. However, 94CTS/5A/GL demonstrated slightly appropriate mechanical behavior in terms of Young’s modulus, maximum tensile strength, and elongation at break. Therefore, 94CTS/5A/GL appeared to be the best compromise the mechanical behavior, the biocidal properties, the biocompatibility, and the surface free energy were prioritized. It is worth noting that dressings obtained by combining A and S (97CTS/2A/S and 94CTS/5A/S) also satisfied most of the criteria, except for the biocidal properties. For this reason, they could be used in late stages of the wound healing process; meanwhile, dressings with GL could be employed in the first ones where the bacterial colonization takes place. In this scenario, a multiple treatment using different dressings to tackle the different phases of the process was envisioned. In conclusion, this research underscores the promise of integrating natural polymers with bioactive agents to create multifunctional materials, addressing current limitations in wound care and offering an innovative approach to regenerative medicine. Future studies should explore *in vivo* applications and clinical trials to validate the efficacy of these films in real-world scenarios.

## Abbreviations

CTS: chitosan
A: aloe vera
S: sorbitol
GL: gluconolactone
*R*_a_: average roughness
*R*_q_: root-mean-square roughness
IFT: surface free energy
IFT (s,P): polar component
IFT (s,D): dispersive component
